# The Role of Preoperative Inflammatory Markers in Pancreatectomy: a Norwegian Nationwide Cohort Study

**DOI:** 10.1007/s11605-023-05726-5

**Published:** 2023-06-15

**Authors:** Mushegh A. Sahakyan, Dyre Kleive, Rachel G. Dille-Amdam, Trond Kjeseth, Kim Waardal, Bjørn Edwin, Linn S. Nymo, Kristoffer Lassen

**Affiliations:** 1 The Intervention Center, Oslo University Hospital, Rikshospitalet, Oslo Norway; 2 Department of Research & Development, Division of Emergencies and Critical Care, Oslo University Hospital, Oslo, Norway; 3 Department of Surgery N1, Yerevan State Medical University After M. Heratsi, Yerevan, Armenia; 4 Department of HPB Surgery, Oslo University Hospital, Rikshospitalet, Oslo Norway; 5 Department of Gastrointestinal Surgery, St. Olavs Hospital, Trondheim University Hospital, Trondheim, Norway; 6 Department of Gastrointestinal Surgery, Stavanger University Hospital, Stavanger, Norway; 7 Department of Acute and Digestive Surgery, Haukeland University Hospital, Bergen, Norway; 8 Institute of Clinical Medicine, Medical Faculty, University of Oslo, Oslo, Norway; 9 Department of Gastrointestinal Surgery, University Hospital of North Norway, Tromsø, Norway; 10 Institute of Clinical Medicine, UiT, the Arctic University of Norway, Tromsø, Norway

**Keywords:** Pancreatectomy, Inflammation, Morbidity, Cancer, Survival

## Abstract

**Background and purpose:**

Preoperative inflammatory markers, such as Glasgow prognostic score, modified Glasgow prognostic score and C-reactive protein to albumin ratio, were shown to be associated with prognosis in patients undergoing pancreatectomy for cancer. However, little is known about their predictive role in a Western population.

**Methods:**

The Norwegian National Registry for Gastrointestinal Surgery (NORGAST) was used to capture all pancreatectomies performed within the study period (November 2015—April 2021). The association between the preoperative inflammatory markers and postoperative outcomes was studied. Their impact on survival was examined in patients operated for pancreatic ductal adenocarcinoma.

**Results:**

A total of 1554 patients underwent pancreatectomy in this period. Glasgow prognostic score, modified Glasgow prognostic score and C-reactive protein to albumin ratio were associated with severe complications (Accordion grade ≥ III) in the univariable but not in the multivariable analysis. C-reactive protein to albumin ratio, but not Glasgow prognostic score and modified Glasgow prognostic score, was linked to survival following pancreatectomy for ductal adenocarcinoma. In the multivariable model, age, neoadjuvant chemotherapy, ECOG score, C-reactive protein to albumin ratio and total pancreatectomy correlated with survival. Also, preoperative C-reactive protein to albumin ratio was significantly associated with survival after pancreatoduodenectomy.

**Conclusions:**

Preoperative Glasgow prognostic score, modified Glasgow prognostic score and C-reactive protein to albumin ratio have no role in predicting the complications after pancreatectomy. C-reactive protein to albumin ratio is a significant predictor for survival in ductal adenocarcinoma, yet its clinical relevance should be explored in conjunction with the pathology parameters and adjuvant therapy.

**Supplementary Information:**

The online version contains supplementary material available at 10.1007/s11605-023-05726-5.

## Introduction

Cancer cells are known to activate systemic inflammatory pathways thereby providing favorable environment for cancer progression, immune evasion, and dissemination.^1–3^ In pancreatic cancer, inflammatory markers such as Glasgow prognostic score (GPS), platelet to lymphocyte ratio and neutrophil to lymphocyte ratio were shown to be associated with prognosis.^4–6^ GPS based on serum C-reactive protein and albumin levels was first introduced as a predictor for treatment outcome in primary unresectable pancreatic cancer.^6^ However, it soon became increasingly used also in patients undergoing pancreatectomy for cancer.^7–9^

Recently, other inflammatory markers derived from serum C-reactive protein and albumin levels, such as modified GPS (mGPS) and C-reactive protein to albumin ratio (CAR), have been reported in the literature.^10,11^ These were considered more sensitive than GPS in terms of predictive qualities, however published results are inconsistent and require further exploration.^7,8,12,13^ Notably, most of the studies comparing different inflammatory markers were conducted in Asia, while only a handful of reports have been published in the Western world.^4,14^ Furthermore, most of the studies come from single centers and are affected by relatively small sample size.

This study aims to examine the association between the preoperative inflammatory markers (GPS, mGPS, CAR), and postoperative outcomes of pancreatectomy in a complete national cohort, as well as their impact on survival in patients operated for pancreatic ductal adenocarcinoma (PDAC).

## Materials and methods

### Study design

This is an observational nationwide cohort study using data collected in the Norwegian National Registry for Gastrointestinal Surgery (NORGAST). This registry covers all surgical (gastrointestinal, hepato-pancreato-biliary) procedures performed in Norway since 2015 including pancreatic resections.^15^ The Norwegian health care system is centralized to a degree where all patients referred for pancreatectomy are operated in one of the five hepato-pancreato-biliary units located at the corresponding public university hospital. Each of these belongs to one of the four independent regional health authorities: South-Eastern, Western, Central and Northern. Data collection, procedure coding, as well as inclusion and exclusion criteria for NORGAST have been meticulously described elsewhere.^16–18^ Information on patient demographics, baseline characteristics (including preoperative GPS, mGPS and CAR), surgical procedures, postoperative outcomes and survival are prospectively registered and updated.

All patients who had undergone pancreatectomy for benign or malignant lesions in the pancreas and periampullary region were included in this study. Study period ranged from November 2015 to April 2021. The association between preoperative inflammatory markers (GPS, mGPS and CAR) and postoperative outcomes (severe complications, relaparotomy, single- and multiorgan failure, 90-day mortality) was examined. Patients without information on preoperative serum albumin, C-reactive protein or tumor histology were excluded from the analysis. The impact of GPS, mGPS and CAR on survival was studied in a subgroup containing only patients with PDAC. Hence, those diagnosed with other histological entities were excluded from the survival analysis. The last follow-up date was May 31st, 2021.

The manuscript was completed in accordance with the Strengthening the Reporting of Observational Studies in Epidemiology (STROBE) statement.^19^ Patients included in NORGAST have given written informed consent for storing their data in the registry. Also, NORGAST holds a data storage license from the Norwegian Data Authority. The current study was approved by the Regional Ethics Committee (2021/ 268,695).

### Definitions

Serum C-reactive protein and albumin levels observed at the last preoperative examination were used for estimating GPS, mGPS and CAR. Patients with normal serum C-reactive protein and albumin levels were defined as having GPS 0, while those with normal serum C-reactive protein level and any albumin level scored mGPS 0. Patients with elevated serum C-reactive protein level (> 10 mg/L) and normal albumin level scored 1 for both GPS and mGPS, while those with normal C-reactive protein level and hypoalbuminemia (< 35 g/L) were graded as GPS 1. Finally, patients with both elevated serum C-reactive protein level and hypoalbuminemia were defined as having GPS/mGPS 2.

Surgical procedures reported in this study included pancreatoduodenectomy, total and distal pancreatectomy, as well as enucleations and other resections. Postoperative complications were defined and classified according to the modified Accordion score.^20^ Complications graded ≥ III were defined as severe. Postoperative mortality was defined as death within 90 days after surgery.^21^ Overall survival was defined as the time between the date of surgery and the date of death from any cause or the date of censoring.

### Statistics

Variables presented are either continuous or categorical. The latter are shown in frequencies (percentages) and analyzed by using the Chi-square and Fisher’s exact test. Normally distributed continuous data are presented with mean (standard deviation), while non-normally distributed (skewed) continuous data are presented with median (range). Student’s t-test and Mann–Whitney U test were used for normally and non-normally distributed continuous data, respectively. A two-tailed *p*-value < 0.05 was considered statistically significant. Parameters that were significant in the univariable analysis were included in the multivariable binary logistic regression model.

The associations between preoperative inflammatory markers and survival were examined by using the log-rank test and the Kaplan–Meier curves were plotted. Survival was described as median (95% confidence interval). Independent predictors for survival were estimated by using the Cox regression model. Parameters significant at p < 0.05 in the univariable analysis were included in the multivariable model with backward selection.

## Results

### Postoperative results

**Fig. 1:**
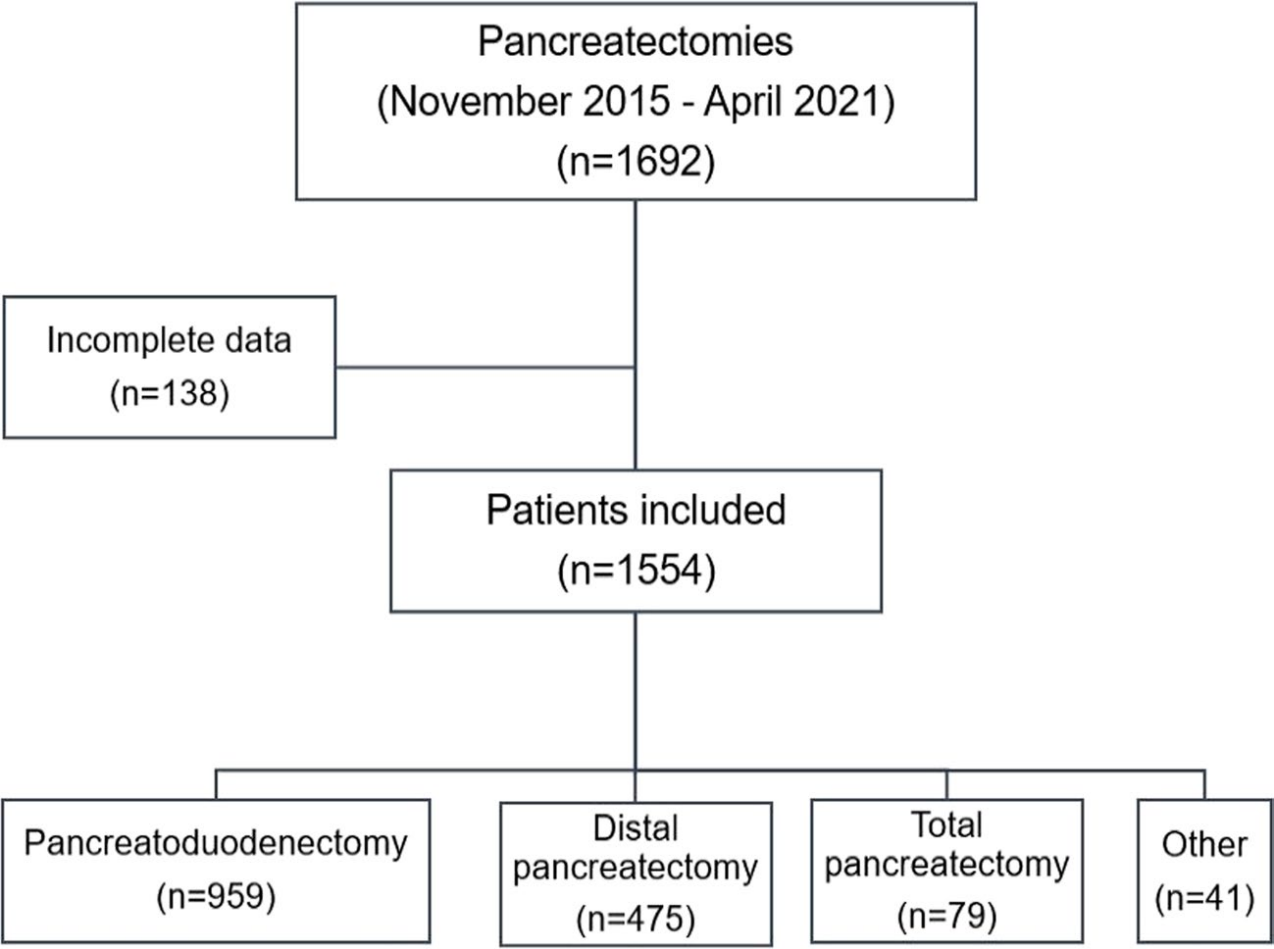
Study flow-chart

A total number of 1554 patients were eligible for the analysis (Fig. 1), of which 959 (61.7%) had undergone pancreatoduodenectomy. All pancreatoduodenectomies and total pancreatectomies were done open, while 324 patients undergoing distal pancreatectomy were operated laparoscopically.

Based on preoperative laboratory findings, 287 and 234 patients scored GPS 1 and mGPS 1 respectively, while 113 scored GPS/mGPS 2. Median CAR was 0.11 (0.02–18.1). Severe complications and 90-day mortality were observed in 483 (31%) and 45 (2.9%) patients, respectively.

GPS, mGPS and CAR significantly correlated with severe complications after pancreatectomy (Table 1). To adjust for confounding, these were included in the multivariable model together with the parameters that were significant in the univariable analysis (gender, body mass index, ECOG score, ASA grade, type of surgical procedure). Multivariable analysis demonstrated that none of the preoperative inflammatory markers were associated with severe complications, unlike gender, body mass index, ECOG score and pancreatoduodenectomy.

**Table 1:** Patient characteristics and perioperative parameters associated with severe complications after pancreatectomy

Parameters	All patients	Severe complications		*p*-value	Multivariable 1^┼^	*p*-value	Multivariable 2^╪^	*p*-value
	*n* = 1554	Yes (*n* = 483)	No (*n* = 1071)		OR (95% CI)		OR (95% CI)	
Age, years, mean (SD) ^¶^	65.8 (11.7)	66 (11.6)	65.7 (11.7)	0.61				
Gender, n (%) ^¶^				0.001		0.001		0.001
Male	818 (53.6%)	293 (62.3%)	525 (49.8%)		1.69 (1.34–2.15)		1.7 (1.35–2.15)	
Female	707 (46.4%)	177 (37.7%)	530 (50.2%)		Reference		Reference	
BMI, kg/m^2^, mean (SD)	25.5 (4.4)	26.1 (4.7)	25.2 (4.2)	0.001	1.05 (1.02–1.08)	0.001	1.05 (1.02–1.08)	0.001
Weight loss, %, mean (SD)	6.5 (7.0)	6.6 (7.1)	6.5 (6.9)	0.76				
Diabetes, n (%)	290 (18.7%)	84 (17.4%)	206 (19.2%)	0.39				
Severe lung disease, n (%)	18	6	12	0.84				
Severe cardiac disease, n (%)	25	10	15	0.33				
Neoadjuvant chemo, n (%)	152 (9.8%)	50 (10.4%)	102 (9.5%)	0.65				
Histology (ductal adenocarcinoma)	606 (39%)	178 (36.8%)	428 (40%)	0.36				
ECOG score, n (%) ^¶^				0.001				
0	962 (63.8%)	267 (56.9%)	695 (66.8%)		Reference		Reference	
1	439 (29.1%)	152 (32.4%)	287 (27.6%)		1.32 (1.02–1.71)	0.036	1.32 (1.02–1.71)	0.036
≥ 2	108 (7.1%)	50 (10.7%)	58 (5.6%)		2.14 (1.37–3.33)	0.001	2.13 (1.37–3.33)	0.001
ASA score ≥ III, n (%)	739 (47.6%)	259 (53.6%)	480 (44.8%)	0.001	1.19 (0.94–1.52)	0.14	1.19 (0.94–1.52)	0.14
GPS, n (%) ^┼^, ^╪^				0.001				
0	1154 (74.3%)	344 (71.2%)	810 (75.6%)		Reference		___________	
1	287 (18.5%)	86 (17.8%)	201 (18.8%)		0.8 (0.57–1.11)	0.18	___________	
2	113 (7.3%)	53 (11%)	60 (5.6%)		1.23 (0.72–2.09)	0.45	___________	
mGPS, n (%) ^┼^, ^╪^				0.001				
0	1207 (77.7%)	361 (74.7%)	846 (79%)		___________		Reference	
1	234 (15.1%)	69 (14.3%)	165 (15.4%)		___________		0.77 (0.54–1.11)	0.16
2	113 (7.3%)	53 (11%)	60 (5.6%)		___________		1.22 (0.71–2.08)	0.47
CAR, median (range)	0.11 (0.02–18.1)	0.12 (0.02–18.1)	0.11 (0.02–10.2)	0.005	1.03 (0.83–1.27)	0.8	1.04 (0.84–1.29)	0.74
Surgical procedure, n (%)				0.008				
Distal pancreatectomy	475 (30.6%)	121 (25.1%)	354 (33.1%)		Reference		Reference	
Pancreatoduodenectomy	959 (61.7%)	324 (67.1%)	635 (59.3%)		1.48 (1.14–1.93)	0.004	1.47 (1.13–1.92)	0.004
Total pancreatectomy	79 (5.1%)	22 (4.6%)	57 (5.3%)		1.16 (0.66–2.04)	0.59	1.15 (0.66–2.02)	0.63

^*¶*^* incomplete data; *^*┼*^* includes GPS in the model; *^*╪*^* includes mGPS in the model*

A total number of 959 patients underwent pancreatoduodenectomy throughout the study period. Preoperative inflammatory parameters were not associated with severe complications, multi-organ failure and relaparotomy but significantly correlated with 90-day mortality, while CAR was linked to single-organ failure after surgery (suppl. Table 1). In the univariable analysis, age, gender and ECOG score were also significant predictors for 90-day mortality following pancreatoduodenectomy (Table 2). In the multivariable regression model, only male gender and ECOG ≥ 1 were associated with 90-day mortality. CAR did was not correlate with single-organ failure in the multivariable analysis (suppl. Table 2). In distal pancreatectomy, GPS ≥ 1 and mGPS ≥ 1 were found to be associated with relaparotomy but not with severe complications (suppl. Table 3). However, these associations were not statistically significant in the multivariable analysis (suppl. Table 4).

**Table 2:** Patient characteristics and perioperative parameters associated with 90-day mortality after pancreatoduodenectomy (backward stepwise regression model)

Parameters	90-day mortality		*p*-value	Multivariable model	*p*-value
	Yes(*n* = 32)	No (*n* = 927)		Odds ratio (95% CI)	
Age, years, mean (SD) ^¶^	71.3 (8.9)	66.8 (10.4)	0.016	1.05 (1.0–1.09)	0.052
Male gender, n (%) ^¶^	25 (78.1%)	497 (53.7%)	0.006	3.43 (1.37–8.58)	0.008
BMI, kg/m^2^, mean (SD)	25.7 (3.7)	25 (4.2)	0.32		
Weight loss, %, mean (SD)	8.9 (6.1)	7.5 (6.8)	0.33		
Diabetes, n (%)	6 (18.8%)	159 (17.2%)	0.81		
Severe lung disease, n (%)	0	13	1.0		
Severe cardiac disease, n (%)	1	12	0.36		
Neoadjuvant chemo, n (%)	4 (12.5%)	113 (12.2%)	1.0		
Histology (ductal adenocarcinoma)	11 (34.4%)	421 (45.4%)	0.37		
ECOG score, n (%) ^¶^			0.004		
0	11 (36.7%)	542 (60.4%)		Reference	
≥ 1	19 (63.3%)	356 (39.6%)		2.47 (1.11–5.47)	0.027
ASA score ≥ III, n (%)	21 (65.6%)	459 (49.5%)	0.07		
GPS, n (%)			0.032		
0	16 (50%)	631 (68.1%)		_______________	-
≥ 1	16 (50%)	296 (31.9%)		_______________	-
mGPS, n (%)			0.043		
0	18 (56.2%)	673 (72.6%)		_______________	-
≥ 1	14 (43.8%)	254 (27.4%)		_______________	-
CAR, median (range)	0.26 (0.02–2.41)	0.12 (0.02–11.7)	0.005	_______________	-

^*¶*^* incomplete data*

### Survival analysis

**Fig. 2:**
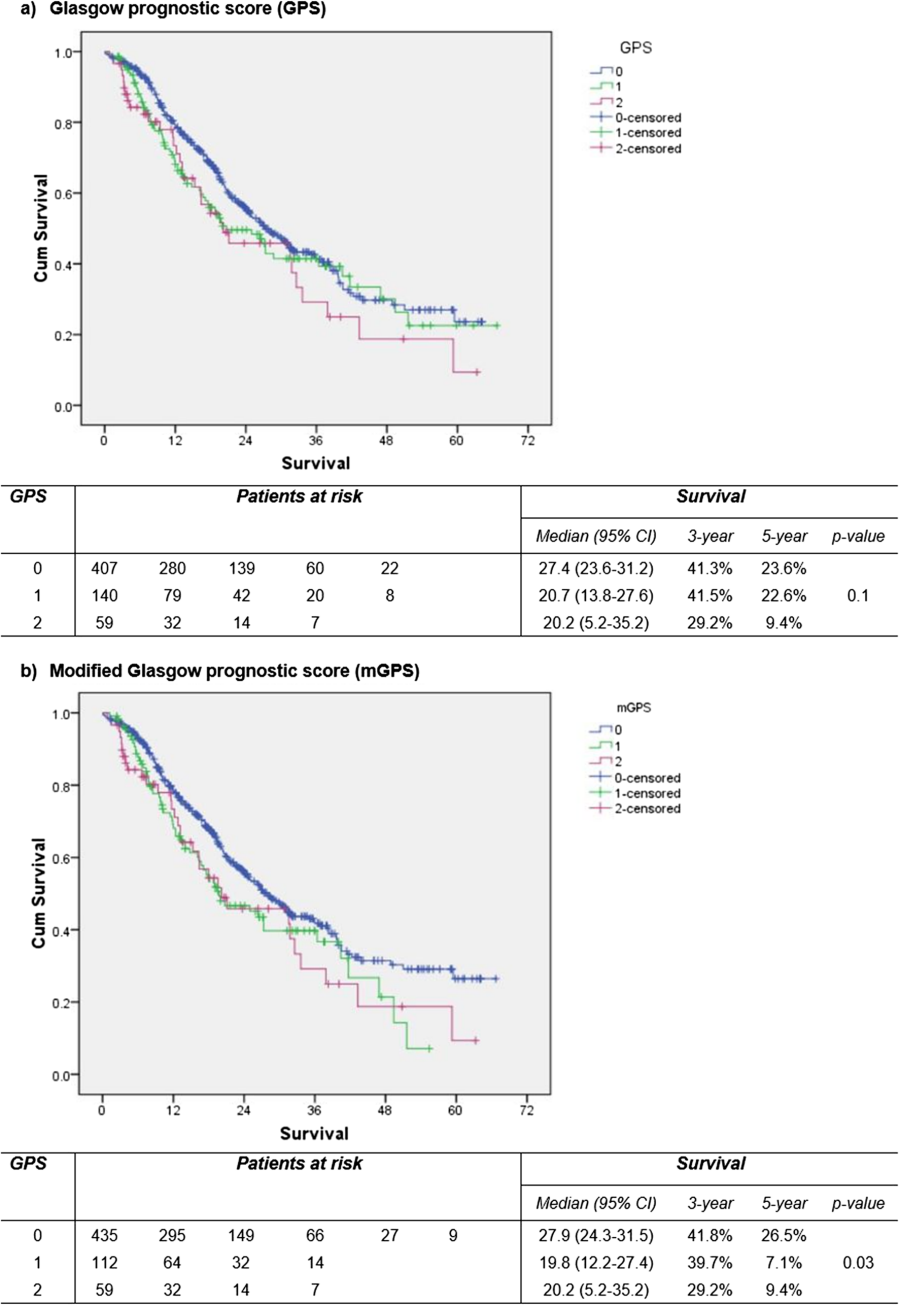
Kaplan–Meier survival plots after pancreatectomy for ductal adenocarcinoma stratified by preoperative Glasgow (**a**) and modified Glasgow (**b**) prognostic scores

After excluding the patients not diagnosed with PDAC, 606 patients were eligible for survival analysis. Kaplan–Meier plots for GPS and mGPS were depicted (Fig. 2). A lower mGPS was associated with longer survival (p = 0.03). Univariable Cox regression analysis identified age, neoadjuvant chemotherapy, ECOG score, ASA grade, CAR, total pancreatectomy and severe complications also to be associated with survival (Table 3). In the multivariable model, older age, neoadjuvant chemotherapy, ECOG ≥ 2, higher CAR and total pancreatectomy were independent negative predictors for survival.

**Table 3:** Uni- and multivariable Cox regression analyses of prognostic factors after pancreatectomy for ductal adenocarcinoma (backward stepwise regression model)

Parameters	Univariable analysis	*p*-value	Multivariable analysis*	*p*-value
	Hazard ratio (95% CI)		Hazard ratio (95% CI)	
Age, years ^¶^	1.02 (1.01–1.03)	0.001	1.02 (1.01–1.03)	0.002
Gender ^¶^				
Male	1.02 (0.81–1.28)	0.87		
Female	Reference			
BMI, kg/m^2^	0.98 (0.95–1.01)	0.1		
Weight loss	1.01 (0.99–1.02)	0.6		
Diabetes	0.98 (0.75–1.3)	0.91		
Severe lung disease	1.17 (0.48–2.82)	0.74		
Severe cardiac disease	0.51 (0.19–1.38)	0.18		
Neoadjuvant chemo	1.49 (1.13–1.97)	0.005	1.51 (1.12–2.03)	0.007
ECOG score ^¶^				
0	Reference		Reference	
1	1.36 (1.06–1.74)	0.014	1.18 (0.91–1.52)	0.21
≥ 2	1.97 (1.31–2.96)	0.001	1.76 (1.16–2.68)	0.01
ASA score ≥ III	1.41 (1.12–1.78)	0.003	_______________	-
GPS				
0	Reference			
1	1.22 (0.93–1.59)	0.16		
2	1.43 (0.98–2.07)	0.063		
mGPS				
0	Reference			
1	1.36 (1.02–1.82)	0.036	_______________	-
2	1.44 (0.99–2.09)	0.049	_______________	-
CAR	1.31 (1.15–1.49)	< 0.001	1.26 (1.11–1.44)	< 0.001
Surgical procedure				
Distal pancreatectomy	Reference		Reference	
Pancreatoduodenectomy	1.08 (0.81–1.44)	0.61	1.01 (0.74–1.36)	0.98
Total pancreatectomy	1.88 (1.18–2.99)	0.008	1.68 (1.03–2.75)	0.04
Severe complications	1.28 (0.99–1.64)	0.049	1.25 (0.97–1.62)	0.08

^*¶*^* incomplete complete*

Four hundred thirty-two patients underwent pancreatoduodenectomy for PDAC. Potential predictors for survival were included in the univariable analysis, which identified age, ECOG score, ASA grade and CAR to be statistically significant (Table 4). In the multivariable analysis, older age, ECOG ≥ 2 and higher CAR were negatively correlated with survival. One hundred thirty-eight patients underwent distal pancreatectomy for PDAC. Preoperative inflammatory markers had no significant impact on survival when analyzed with other perioperative parameters (suppl. Table 5).

**Table 4:** Uni- and multivariable Cox regression analyses of prognostic factors in patients undergoing pancreatoduodenectomy for ductal adenocarcinoma

Parameters	Univariable analysis	*p*-value	Multivariable analysis	*p*-value
	Hazard ratio (95% CI)		Hazard ratio (95% CI)	
Age, years ^¶^	1.03 (1.01–1.05)	0.001	1.025 (1.01–1.04)	0.006
Gender ^¶^				
Male	1.08 (0.83–1.42)	0.56		
Female	Reference			
BMI, kg/m^2^	0.99 (0.96–1.03)	0.58		
Weight loss	1.01 (0.99–1.03)	0.65		
Diabetes	1.04 (0.75–1.46)	0.79		
Severe lung disease	1.35 (0.5–3.62)	0.56		
Severe cardiac disease	0.39 (0.097–1.59)	0.19		
Neoadjuvant chemo	1.29 (0.93–1.79)	0.13		
ECOG score ^¶^				
0	Reference		Reference	
1	1.32 (0.99–1.77)	0.062	1.19 (0.89–1.62)	0.24
≥ 2	1.79 (1.1–2.94)	0.019	1.67 (1.02–2.79)	0.04
ASA score ≥ III	1.46 (1.11–1.92)	0.006	1.14 (0.84–1.55)	0.39
GPS				
0	Reference			
1	1.13 (0.83–1.56)	0.44		
2	1.38 (0.91–2.11)	0.13		
mGPS				
0	Reference			
1	1.35 (0.97–1.89)	0.078		
2	1.43 (0.94–2.18)	0.094		
CAR	1.29 (1.11–1.49)	0.001	1.24 (1.06–1.45)	0.008
Severe complications	1.12 (0.83–1.50)	0.47		

^*¶*^* incomplete data*

### Subgroup analysis

Distribution and clinical relevance of preoperative inflammatory markers were studied in a subgroup of patients receiving neoadjuvant chemotherapy. The latter was applied in 152 (9.8%) patients. The rate of GPS I/II among those with and without neoadjuvant chemotherapy was 21.7% / 5.9% vs 18.1% / 7.4% (*p* = 0.48), respectively. mGPS I / II were observed in 14.9% / 7.4% of patients with and 16.4% / 5.9% of patients without neoadjuvant chemotherapy (*p* = 0.73). There were no statistically significant differences in median CAR values among those with and without neoadjuvant chemotherapy – 0.11 (0.02–5.42) vs 0.11 (0.02–18.07) (*p* = 0.68), respectively. No statistically significant correlations were seen between preoperative inflammatory markers and outcome parameters (such as postoperative complications, 90-day mortality and survival) after neoadjuvant chemotherapy.

## Discussion

Our findings indicate that CAR is a significant predictor for survival in patients undergoing pancreatectomy for PDAC. In contrast, GPS and mGPS have no predictive role in these patients. This is in line with the recent publications from Europe and Asia.^14,22–24^ Van Wijk and co-workers analyzed 163 patients with PDAC suggesting that CAR (categorized as < 0.2 and ≥ 0.2) outperforms mGPS as survival predictor.^14^ Ikuta et al. analyzed CAR together with mGPS and other inflammatory markers demonstrating significantly higher sensitivity of CAR in terms of survival prediction.

Some studies have reported the ability of preoperative inflammatory markers to predict postoperative complications after major abdominal surgery.^25,26^ Knight and co-workers demonstrated that GPS is associated with postoperative outcomes following pancreatectomy.^27^ However, these findings were not confirmed in our study, as preoperative inflammatory makers failed to maintain their predictive relevance when adjusted for confounders. Furthermore, the associations with postoperative outcomes remained non-significant after stratifying by the type of surgical procedure performed. Male gender, obesity and ECOG performance status were identified as preoperative parameters that were independent predictors for postoperative complications after pancreatectomy. All of these factors reflecting patients’ functional capacities are well-known risk factors for postoperative complications in major abdominal surgery and pancreatectomy specifically.^28–30^ Pancreatoduodenectomy was expectedly associated with a higher likelihood of complications compared with the distal pancreatectomy given the extent and procedure-related risks of the former.

One major difference between this study and other reports in the literature is that the present study was based on the analysis of a complete nationwide cohort. This allowed for encompassing all patients that had undergone pancreatectomy in Norway throughout the study period without any selection bias. Furthermore, survival data were prospectively updated and available in all patients at the last follow-up. Another major difference is that performance of preoperative inflammatory parameters was studied for different surgical procedures (pancreatoduodenectomy and distal pancreatectomy). This aspect has received little attention before as studies normally report pooled data without distinguishing the type of pancreatic resection. This study suggests that the impact of CAR on survival is highly relevant in pancreatoduodenectomy, but negligible in distal pancreatectomy.

This study has several limitations worth mentioning. First and foremost, given the specific design of NORGAST a limited number of variables was included in the analysis. One should remember that this registry is primarily designed for covering postoperative results rather than oncologic outcomes. As a result, pathology-related parameters are not registered although data on tumor histology was retrieved retrospectively. Hence, predictive relevance of CAR needs to be validated in a cohort including specific information on pathology parameters. Second, NORGAST contains no information on adjuvant therapy, disease recurrence and recurrence-free survival. Given the importance of these parameters and the fact that their association with preoperative inflammatory markers has been previously reported, ^8,23,31^ it would be desirable to include them in the analysis. Third, some of the baseline parameters were incomplete, although in a very small proportion of cases (2–3%).

## Conclusion

Preoperative inflammatory markers appear to have no role in predicting short term postoperative outcomes of pancreatectomy. CAR is, however, a significant predictor for survival in patients with PDAC, especially those undergoing pancreatoduodenectomy. Neither GPS nor mGPS have any such role. The predictive role of CAR should be tested together with the pathology-based parameters and adjuvant therapy in a large nationwide dataset allowing for better understanding of its clinical relevance.

## Supplementary Information

Below is the link to the electronic supplementary material.

Supplementary file1 (DOCX 33.7 KB)

## Data Availability

The data that support the findings of this study are available from NORGAST upon application. Restrictions apply to the availability of these data, which were used under license for this study.
