# Supplementary material for: The Role of Preoperative Inflammatory Markers in Pancreatectomy: a Norwegian Nationwide Cohort Study
Source: J Gastrointest Surg. 2023 Jun 15;27(8):1650–9. doi: 10.1007/s11605-023-05726-5 (PMC10412490; doi:10.1007/s11605-023-05726-5)
Supplement: Supplementary file 1 — Supplementary file1 (DOCX 33.7 KB) [file 11605_2023_5726_MOESM1_ESM.docx]

**Supplementary table 1. Preoperative inflammatory markers and postoperative results following pancreatoduodenectomy (a – GPS, mGPS; b – CAR).**

**a)**

| **Parameters** | **GPS^┼^** | | | **mGPS^╪^** | | |
| --- | --- | --- | --- | --- | --- | --- |
|  | **0**  **(n=647)** | **1**  **(n=221)** | **2**  **(n=91)** | **0**  **(n=691)** | **1**  **(n=177)** | **2**  **(n=91)** |
| Severe complications, n (%) | 217 (33.6%) | 67 (30.3%) | 40 (43.9%) | 229 (33.1%) | 55 (31.1%) | 40 (43.9%) |
| Single-organ failure, n (%) | 37 | 14 | 11 | 39 | 12 | 11 |
| Multi-organ failure, n (%) | 12 | 7 | 5 | 13 | 6 | 5 |
| Relaparotomy, n (%) | 95 (14.7%) | 27 (12.2%) | 16 (17.6%) | 101 (14.6%) | 21 (11.9%) | 16 (17.6%) |
| 90-day mortality, n (%) ***** | 16 | 9 | 7 | 18 | 7 | 7 |

**^┼^**Glasgow prognostic score; **^╪^** modified Glasgow prognostic score; * statistically significant difference

**b)**

| **Parameters** | **Severe complications** | | **Single-organ failure*** | | **Multi-organ failure** | | **Relaparotomy** | | **90-day mortality*** | |
| --- | --- | --- | --- | --- | --- | --- | --- | --- | --- | --- |
|  | **Y=324** | **N=635** | **Y=62** | **N=897** | **Y=24** | **N=935** | **Y=138** | **N=821** | **Y=32** | **N=927** |
| CAR**^¶^**, median (range) | 0.13 (0.02-11.7) | 0.12 (0.02-5.03) | 0.16 (0.02-3.85) | 0.12 (0.02-11.7) | 0.26 (0.02-2.41) | 0.12 (0.02-11.7) | 0.14 (0.02-4.8) | 0.12 (0.02-11.7) | 0.26 (0.02-2.41) | 0.12 (0.02-11.7) |

***^¶^*** *CRP-albumin ratio**; * statistically significant difference*

**Supplementary table 2. Patient characteristics and perioperative parameters associated with single-organ failure after pancreatoduodenectomy (backward stepwise regression model).**

| **Parameters** | *Univariable model* | | **p-value** |  | *Multivariable model* | **p-value** |
| --- | --- | --- | --- | --- | --- | --- |
|  | **Single-organ failure** | |  |  | **Single-organ failure** |  |
|  | **Yes(n=62)** | **No (n=897)** |  |  | **Odds ratio (95% CI)** |  |
| Age, years, mean (SD) ^¶^ | 67.9 (10.8) | 66.9 (10.3) | 0.43 |  |  |  |
| Male gender, n (%) ^¶^ | 41 (66.1%) | 481 (53.7%) | 0.057 |  |  |  |
| BMI, kg/m^2^, mean (SD) | 26.2 (4.1) | 24.9 (4.2) | 0.018 |  | 1.07 (1.01-1.13) | 0.026 |
| Weight loss, %, mean (SD) | 6.5 (6.5) | 7.6 (6.8) | 0.37 |  |  |  |
| Diabetes, n (%) | 8 (12.9%) | 157 (17.5%) | 0.35 |  |  |  |
| Severe lung disease, n (%) | 1 (1.6%) | 12 (1.3%) | 0.58 |  |  |  |
| Severe cardiac disease, n (%) | 3 (4.8%) | 10 (1.1%) | 0.046 |  | _______________ | - |
| Neoadjuvant chemo, n (%) | 9 (14.5%) | 108 (12%) | 0.57 |  |  |  |
| Histology (ductal adenocarcinoma) | 19 (30.6%) | 413 (46%) | 0.1 |  |  |  |
| ECOG score, n (%) ^¶^ |  |  | 0.35 |  |  |  |
| 0 | 32 (54.2%) | 521 (60%) |  |  |  |  |
| ≥ 1 | 27 (45.8%) | 348 (40%) |  |  |  |  |
| ASA score ≥ III, n (%) | 41 (66.1%) | 439 (48.9%) | 0.009 |  | 2.09 (1.2 – 3.64) | 0.009 |
| GPS^┼^, n (%) |  |  | 0.07 |  |  |  |
| 0 | 37 (59.7%) | 610 (68%) |  |  |  |  |
| 1 | 14 (22.6%) | 207 (23.1%) |  |  |  |  |
| 2 | 11 (17.7%) | 80 (8.9%) |  |  |  |  |
| mGPS^╪^, n (%) |  |  | 0.062 |  |  |  |
| 0 | 39 (62.9%) | 652 (72.7%) |  |  |  |  |
| 1 | 12 (19.4%) | 165 (18.4%) |  |  |  |  |
| 2 | 11 (17.7%) | 80 (8.9%) |  |  |  |  |
| CAR^⁰^, median (range) | 0.16 (0.02-3.85) | 0.12 (0.02-11.7) | 0.017 |  | _______________ | - |

*^¶^ incomplete data; ^┼^Glasgow prognostic score; ^╪^modified Glasgow prognostic score;* ^⁰^*CRP-albumin ratio.*

**Supplementary table 3. Preoperative inflammatory markers and postoperative results following distal pancreatectomy (a – GPS, mGPS; b – CAR).**

**a)**

| **Parameters** | **GPS^┼^** | | **mGPS^╪^** | |
| --- | --- | --- | --- | --- |
|  | **0**  **(n=421)** | **≥ 1**  **(n=54)** | **0**  **(n=424)** | **≥ 1**  **(n=51)** |
| Severe complications, n (%) ^⁰^ | 103 | 18 | 105 | 16 |
| Relaparotomy, n (%) ***** | 21 | 7 | 21 | 7 |

**^┼^**Glasgow prognostic score; **^╪^** modified Glasgow prognostic score; ^⁰^ no statistically significant difference; * statistically significant difference

**b)**

| **Parameters** | **Severe complications***^┼^* | | **Relaparotomy***^┼^* | |
| --- | --- | --- | --- | --- |
|  | **Y=121** | **N=354** | **Y=28** | **N=447** |
| CAR**^¶^**, median (range) | 0.08 (0.02-4.57) | 0.07 (0.02-10.2) | 0.08 (0.02-4.57) | 0.07 (0.02-10.2) |

***^¶^*** *CRP-albumin ratio; ^┼^ no statistically significant difference*

**Supplementary table 4. Uni- and multiavariable (backward stepwise regression) analyses of patient characteristics and perioperative parameters associated with relaparotomy after distal pancreatectomy.**

|  | *Univariable model* | | |  | *Multivariable model* | |
| --- | --- | --- | --- | --- | --- | --- |
| **Parameters** | **Relaparotomy** | | **p-value** |  | **Relaparotomy** | **p-value** |
|  | **Yes(n=28)** | **No (n=447)** |  |  | **Odds ratio (95% CI)** |  |
| Age, years, mean (SD) ^¶^ | 62.7 (11.7) | 64.2 (13.6) | 0.57 |  |  |  |
| Male gender, n (%) ^¶^ | 21 | 229 | 0.015 |  | _______________ | - |
| BMI, kg/m^2^, mean (SD) | 28.9 (7.1) | 26.2 (4.4) | 0.003 |  | 1.06 (1.01-1.11) | 0.033 |
| Weight loss, %, mean (SD) | 6.5 (9.8) | 4.1 (6.3) | 0.16 |  |  |  |
| Diabetes, n (%) | 4 | 91 | 0.44 |  |  |  |
| Severe lung disease, n (%) | 0 | 2 | 1.0 |  |  |  |
| Severe cardiac disease, n (%) | 0 | 10 | 1.0 |  |  |  |
| Neoadjuvant chemo, n (%) | 0 | 18 | 0.62 |  |  |  |
| Histology (ductal adenocarcinoma) | 10 | 128 | 0.5 |  |  |  |
| ECOG score, n (%) ^¶^ |  |  | 0.001 |  |  |  |
| 0 | 10 | 325 |  |  | reference |  |
| ≥ 1 | 17 | 114 |  |  | _______________ | - |
| ASA score ≥ III, n (%) | 13 | 184 | 0.58 |  |  |  |
| GPS^┼^, n (%) |  |  | 0.029 |  |  |  |
| 0 | 21 | 400 |  |  | reference |  |
| ≥ 1 | 7 | 47 |  |  | 0.46 (0.17-1.23) | 0.12 |
| mGPS^╪^, n (%) |  |  | 0.022 |  |  |  |
| 0 | 21 | 403 |  |  | reference |  |
| ≥ 1 | 7 | 44 |  |  | _______________ | - |
| CAR^⁰^, median (range) | 0.08 (0.02-4.57) | 0.07 (0.02-10.2) | 0.54 |  |  |  |

*^¶^ incomplete data; ^┼^Glasgow prognostic score; ^╪^modified Glasgow prognostic score;* ^⁰^*CRP-albumin ratio.*

**Supplementary table 5. Uni- and multivariable Cox regression analyses of prognostic factors in patients undergoing distal pancreatectomy for ductal adenocarcinoma.**

| **Parameters** | **Univariable analysis** | **p-value** | **Multivariable analysis** | **p-value** |
| --- | --- | --- | --- | --- |
|  | **Hazard ratio (95% CI)** |  | **Hazard ratio (95% CI)** |  |
| Age, years ^¶^ | 1.01 (0.98-1.04) | 0.38 |  |  |
| Gender ^¶^ |  |  |  |  |
| Male | 0.87 (0.52-1.45) | 0.59 |  |  |
| Female | Reference |  |  |  |
| BMI, kg/m^2^ | 0.92 (0.85-0.99) | 0.02 | 0.91 (0.84-0.98) | 0.012 |
| Weight loss | 1.02 (0.96-1.08) | 0.52 |  |  |
| Diabetes | 0.7 (0.36-1.39) | 0.31 |  |  |
| Severe cardiac disease | 1.53 (0.37-6.34) | 0.56 |  |  |
| Neoadjuvant chemo | 2.22 (0.93-5.31) | 0.07 |  |  |
| ECOG score ^¶^ |  |  |  |  |
| 0 | Reference |  | reference |  |
| ≥ 1 | 2.29 (1.36-3.88) | 0.002 | 2.2 (1.27-3.81) | 0.005 |
| ASA score ≥ III | 1.14 (0.69-1.90) | 0.61 |  |  |
| GPS*^┼^* |  |  |  |  |
| 0 | Reference |  | reference |  |
| ≥ 1 | 1.97 (1.06-3.65) | 0.03 | 1.3 (0.41-4.17) | 0.66 |
| mGPS*^╪^* |  |  |  |  |
| 0 | Reference |  |  |  |
| ≥ 1 | 1.78 (0.94-3.36) | 0.08 |  |  |
| CAR^⁰^ | 2.61 (1.32-5.19) | 0.006 | 1.7 (0.45-6.46) | 0.44 |
| Severe complications | 1.62 (0.94-2.8) | 0.08 |  |  |

*^¶^ incomplete data; ^┼^Glasgow prognostic score; ^╪^modified Glasgow prognostic score;* ^⁰^*CRP-albumin ratio.*
